# Senolytic therapy to modulate the progression of Alzheimer’s Disease (SToMP-AD) – Outcomes from the first clinical trial of senolytic therapy for Alzheimer’s disease

**DOI:** 10.21203/rs.3.rs-2809973/v1

**Published:** 2023-04-24

**Authors:** Mitzi M. Gonzales, Valentina R. Garbarino, Tiffany Kautz, Juan Pablo Palavicini, Marisa Lopez-Cruzan, Shiva Kazempour Dehkordi, Julia Mathews, Habil Zare, Peng Xu, Bin Zhang, Crystal Franklin, Mohamad Habes, Suzanne Craft, Ronald C. Petersen, Tamara Tchkonia, James Kirkland, Arash Salardini, Sudha Seshadri, Nicolas Musi, Miranda E. Orr

**Affiliations:** 1 Glenn Biggs Institute for Alzheimer’s & Neurodegenerative Diseases, University of Texas Health Science Center at San Antonio, San Antonio, TX, USA; 2 Department of Neurology, University of Texas Health Science Center at San Antonio, San Antonio, TX, USA; 3 Department of Medicine, University of Texas Health Science Center at San Antonio, San Antonio, TX, USA; 4 Department of Psychiatry, University of Texas Health Science Center at San Antonio, San Antonio, TX, USA; 5 Department of Cell Systems and Anatomy, University of Texas Health Science Center at San Antonio, San Antonio, TX, USA; 6 Department of Genetics and Genomic Sciences, Icahn School of Medicine at Mount Sinai, New York, NY, USA; 7 Mount Sinai Center for Transformative Disease Modeling, Icahn School of Medicine at Mount Sinai, New York, NY, USA; 8 Research Imaging Institute, University of Texas Health Science Center at San Antonio, San Antonio, TX, USA; 9 Department of Radiology, University of Texas Health Science Center at San Antonio, San Antonio, TX, USA; 10 Department of Internal Medicine Section on Gerontology and Geriatric Medicine, Wake Forest School of Medicine, Winston-Salem, NC, USA; 11 Department of Neurology, Mayo Clinic, Rochester, MN, USA; 12 Department of Physiology and Biomedical Engineering, Mayo Clinic, Rochester, MN, USA; 13 Department of Internal Medicine, Mayo Clinic, Rochester, MN, USA; 14 Department of Neurology, Boston University School of Medicine, Boston, MA, USA; 15 Department of Geriatric Medicine, Cedars-Sinai Medical Center, Los Angeles, CA, USA

## Abstract

Cellular senescence has been identified as a pathological mechanism linked to tau and amyloid beta (Aβ) accumulation in mouse models of Alzheimer’s disease (AD). Clearance of senescent cells using the senolytic compounds dasatinib (D) and quercetin (Q) reduced neuropathological burden and improved clinically relevant outcomes in the mice. Herein, we conducted a vanguard open-label clinical trial of senolytic therapy for AD with the primary aim of evaluating central nervous system (CNS) penetrance, as well as exploratory data collection relevant to safety, feasibility, and efficacy. Participants with early-stage symptomatic AD were enrolled in an open-label, 12-week pilot study of intermittent orally-delivered D+Q. CNS penetrance was assessed by evaluating drug levels in cerebrospinal fluid (CSF) using high performance liquid chromatography with tandem mass spectrometry. Safety was continuously monitored with adverse event reporting, vitals, and laboratory work. Cognition, neuroimaging, and plasma and CSF biomarkers were assessed at baseline and post-treatment. Five participants (mean age: 76±5 years; 40% female) completed the trial. The treatment increased D and Q levels in the blood of all participants ranging from 12.7 to 73.5 ng/ml for D and 3.29–26.30 ng/ml for Q. D levels were detected in the CSF of four participants ranging from 0.281 to 0.536 ng/ml (t(4)=3.123, p=0.035); Q was not detected. Treatment was well-tolerated with no early discontinuation and six mild to moderate adverse events occurring across the study. Cognitive and neuroimaging endpoints did not significantly differ from baseline to post-treatment. CNS levels of IL-6 and GFAP increased from baseline to post-treatment (t(4)=3.913, p=008 and t(4)=3.354, p=0.028, respectively) concomitant with decreased levels of several cytokines and chemokines associated with senescence, and a trend toward higher levels of Aβ42 (t(4)=−2.338, p=0.079). Collectively the data indicate the CNS penetrance of D and provide preliminary support for the safety, tolerability, and feasibility of the intervention and suggest that astrocytes and Aβ may be particularly responsive to the treatment. While early results are promising, fully powered, placebo-controlled studies are needed to evaluate the potential of AD modification with the novel approach of targeting cellular senescence.

## Introduction

Alzheimer’s disease (AD) is the most prevalent cause of dementia, a devastating condition that affects over 35 million individuals worldwide^1^. Historically, drug development for the indication of AD has been among the slowest, most expensive, and least successful with a failure rate of over 99%^2^. Fortunately, recent years have seen the development of disease modifying drugs capable of removing abnormal aggregations of amyloid beta (Aβ) from the brain^3^. Despite these successes, the anti-amyloid drugs have only yielded modest clinical results, spurring consideration of new drug targets and combination treatments^3,4^.

The majority of individuals with AD present with multiple etiological contributors to dementia^5^, suggesting that therapeutic targets beyond Aβ and tau deposition may have a role in treatment. Towards this end, our preclinical research has highlighted cellular senescence as a mechanism that may underlie pathological tau accumulation^6,7^. Cellular senescence is a complex stress response triggered by various stimuli, including macromolecular damage (such as DNA damage), proteotoxic stress, oncogene activation, reactive metabolites, mitochondrial dysfunction, and infections, among others^8^. The stress response leads to a change in cell fate whereby senescent cells enter a near-permanent cell cycle arrest mediated through tumor suppressive pathways^9^. Senescent cells also acquire a senescence-associated secretory phenotype (SASP)^10,11^. The SASP is comprised of cytokines, chemokines, growth factors, and extracellular matrix re-modeling components, which can spread in a paracrine manner and propagate the senescent phenotype to neighboring cells^8,12^. In the context of aging and neurodegenerative disease, senescent cell accumulation has been identified in multiple cell types within the central nervous system, including neurons^6,7,13,14^, astrocytes^15,16^, microglia^17,18^, oligodendrocyte precursor cells^19^, and endothelial cells^20^.

Experimental evidence to support the role of cellular senescence in AD neuropathology has been provided by preclinical trials employing senolytics. Senolytics are pharmacological agents which selectively ablate senescent cells and were first identified through interrogation of the senescent cell anti-apoptotic pathways (SCAPs)^21^. At present, dasatinib (D), a tyrosine-kinase inhibitor that is FDA-approved for chronic myeloid leukemia (CML) and acute lymphoblastic leukemia (ALL)^22^, and quercetin (Q), a natural plant-based flavonoid with anti-inflammatory, antioxidant, and antineoplastic properties^23^, are the best characterized senolytics^8^. When combined, D+Q has been shown to selectively clear senescent cells in culture in both humans and animal models^8,24,25^. In preclinical trials of murine models, D+Q has ameliorated multiple chronic age-related conditions; we previously reported the first evidence to support the potential therapeutic efficacy of D+Q for neurodegenerative disease. Within four tau transgenic mouse models, we found that biweekly administration of D+Q relative to placebo resulted in a 35% reduction in cortical NFT accumulation, which correlated with reduced cortical brain atrophy and restored aberrant cerebral blood flow^6^. Other research teams confirmed the association between tau and senescence^18^ and the effective clearance of senescent cells using D+Q in an Aβ producing mouse model^19^.

Given the compelling evidence provided by preclinical research^6,19^, coupled with the encouraging safety profiles reported in human studies of D+Q for other disease indications^26,27^, we conducted the first clinical trial of senolytic therapy for AD. The aim of the study was to evaluate penetration of D and Q in the central nervous system by performing mass spectrometry on cerebral spinal fluid (CSF) collected prior to treatment and within 80 to 150 minutes of the final study drug dose. We further aimed to collect data on secondary outcomes including safety and feasibility, target engagement of the senolytic compounds, AD CSF and plasma biomarkers, and cognition, neuroimaging, and functional status. We enrolled five participants with early-stage symptomatic AD in an open-label 12-week intervention of intermittent senolytic therapy and provide the first report of the trial outcomes.

## Methods

The study is an open-label single-site pilot study of 12-week intermittent senolytic therapy in older adults with early-stage AD with the primary aim of evaluating the central nervous system penetrance of D and Q (NCT04063124)^28^. Secondary trial aims were to 1) evaluate target engagement of D+Q by examining changes markers associated with cellular senescence and the SASP; 2) assess the safety and tolerability of the intervention; 3) examine pre- to post-treatment changes in cognition and functional status; and 4) assess changes in neuroimaging and biofluid markers of AD and related dementias (ADRD). The study was conducted in adherence with the Guideline for Good Clinical Practice and the protocol was approved by the local institutional review board. All participants provided written informed consent with appropriate legal representation for individuals lacking capacity to consent.

### Participants:

Eligibility for the study included adults aged 65 years and over with a diagnosis of AD based on the criteria for the National Institute on Aging-Alzheimer’s Association^29^ and a Global Clinical Dementia Rating (CDR) Scale score of 1^30^. Anticholinesterase inhibitors and/or memantine use were allowed following a minimum of a three-month stabilization period. Full eligibility criteria were applied as described in Gonzales et al^28^.

### Study Design:

**Figure 1:**
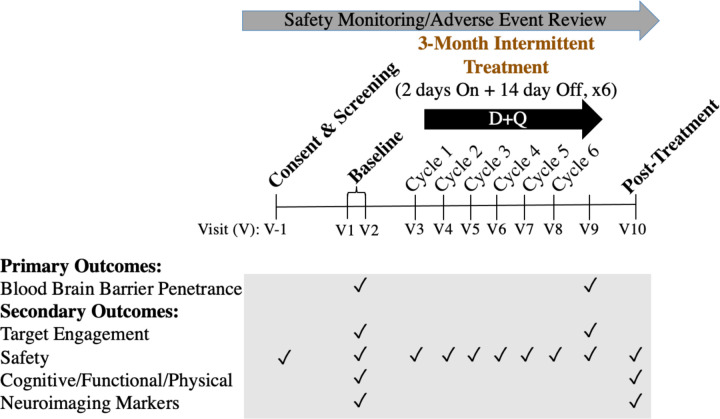
Study Design and Timeline. Modified from Gonzales et al., 2021^28^. Primary outcomes were to assess blood-brain barrier penetrance of the senolytic drugs Dasatinib (D) and Quercetin (Q) (D+Q). Secondary outcomes explored target engagement, safety, functional outcomes and neuroimaging markers.

As previously described^28^, the study protocol included completion of 11 study visits over a period of 20 to 24 weeks (Figure 1). Following obtainment of written informed consent, study candidates completed an in-person screening visit consisting of a blood draw, vital signs, anthropomorphic measurements, physical and neurological examination, medical history and concomitant medication reviews, cognitive screening assessments (CDR^30^ and Montreal Cognitive Assessment (MoCA)^31^), and electrocardiogram (ECG). Following confirmation of study eligibility, participants completed two baseline assessment visits consisting of a fasting blood draw and lumbar puncture (Baseline Visit 1) and assessments of cognition, functional status, and an optional brain MRI (Baseline Visit 2). In response to the onset of the COVID-19 pandemic, the protocol was modified to include confirmation of a negative real-time reverse transcriptase–polymerase chain reaction (rRT-PCR) test within 72 hours of the first study drug administration, and COVID-19 symptom and exposure screenings were conducted across the study. The first study drug administration visit occurred within 3 to 10 days of the second baseline visit. Study drugs, 100mg of D (one 100mg capsule, Sprycel, Bristol Meyers Squibb) and 1000mg of Q (four 250 mg capsules, Thorne Research) were administered consecutively for two days followed by a 13- to 15-day study drug holiday across a total of six cycles (IND #143945). On the first day of each cycle, participants reported to the study site for safety assessments and drug dispensing. Within 80 to 150 minutes of the administration of final study drug dose, participants underwent a fasting blood draw and lumbar puncture. The assessment procedures administered at Baseline Visit 2 were repeated within 3–10 days of the final study drug dose. D+Q were administered under IND # 143945–0006 (to N.M).

### Safety and Adherence:

Vital signs, concomitant medications, and adverse events were reviewed at each study visit. Safety labs, including complete blood count (CBC) with differentials and comprehensive metabolic panel (CMP) with liver and lipid panels, were conducted at Visits 1, 4, 5, 6, 8, and 9. Prothrombin time/partial thromboplastin time/international normalized ratio (PT/PTT/INR) was assessed at Visits 1 and 8 and hemoglobin A1c (HbA1c) was evaluated at Visits 1 and 9. Electrocardiogram was conducted at Visits 1, 4, 6, 8, and 11. The study was monitored by an independent data and safety monitoring board, who reviewed the safety data on an annual basis. Adherence was assessed by the total number of doses completed, counted by administrations in clinic, home diary records, and pill bottle review.

### Cognitive and Functional Outcomes:

The pre-specified cognitive outcomes of interest were pre-to post-treatment changes on the MoCA^31^ and CDR Sum of Boxes (SOB)^30^. Additional cognitive assessments included the Weschler Memory Scale Fourth Edition (WMS-IV) Logical Memory^32^, Benson Figure^33^, Trail Making Test Parts A&B^33^, Number Span Test^33^, Category Fluency^34^, Phonemic Fluency^34^, Boston Naming Test^35^, and the Hopkins Verbal Learning Test Revised (HVLT-R)^36^. Neuropsychiatric symptoms were assessed using the self-reported Geriatric Depression Scale 15-Item (GDS-15) and informant-reported Neuropsychiatric Inventory (NPI)^33^. Functional status was evaluated using the informant-reported Lawton IADL form^37^ and as part of the CDR.

### Brain MRI:

Brain MRI was conducted at the UTHSCSA Research Imaging Institute on a 3-Tesla Siemens Trio scanner. The imaging protocol consisted of a localizer scan, high-resolution 3-dimensional T1-weighted structural series scan, a T2-weighted fluid attention inversion recovery (FLAIR) scan, a diffusion-weighted scan, and a gradient echo scan. Pre- and post-treatment structural scans were spatially coregistered using rigid-body registration, followed by nonlinear registration and multi-atlas based neuroanatomic parcellation, ^38–40^ to quantify total brain and hippocampal volume and grey and white matter density normalized to intracerebroventricular volume (ICV) from four of the five study participants.

### Blood Draws and Lumbar Punctures:

Blood plasma and CSF samples for research purposes were collected according to established procedures^41^. Briefly, blood was collected under fasting conditions via venipuncture in a plasma EDTA vacutainer tube (BD, Franklin Lakes, NJ), inverted 5–10 times, and centrifuged at 2000 x *g* for 10 minutes at room temperature. Plasma was aliquoted and stored at −80°C within 2 hours of collection. CSF was also collected under fasting conditions using a 24-gauge atraumatic Sprotte spinal needle under gravity flow (Teleflex, Morrisville, NC). CSF was collected into a sterile polypropylene tube (Rose Scientific, Alberta, CA), which was centrifuged at 2000 x *g* for 10 minutes at room temperature. CSF was aliquoted and stored at −80°C within 2 hours of collection.

### Assays

#### Drug Concentrations:

Pre- and post-treatment D and Q concentrations in blood and CSF were quantified via High Performance Liquid Chromatography (HPLC) with Tandem Mass Spectrometry detection (MS/MS) method (HPLC/MS/MS) at the UTHSCSA Biological Psychiatry Analytical Lab. Analytical solutions were prepared with Milli-Q Plus water (Millipore Sigma, EMD Millipore, Billerica, MA). D and Q analytical standards were obtained from Sigma (Sigma-Aldrich Corp., St. Louis, MO) and their metabolites (dasatinib n-oxide and 4-o-methyl quercetin) and internal standards (IS) from Cayman (Cayman Chemical, Ann Arbor, MI). All other chemicals were HPLC analytical grade and purchased from Fisher Scientific (Thermo Fisher Scientific, Waltham, MA). Tandem mass spectrometry was performed using a Shimadzu 8045 Triple Quadrupole mass spectrometer (Shimadzu Scientific Instruments, Inc., Houston, TX). The lower limit of detection (LOD) was estimated to be 0.3 ng/ml for D, Q, and their metabolites in plasma and CSF, except for D in CSF, which was estimated to be 0.025 ng.ml. The lower limit of quantitation (LOQ) for D, Q and their metabolites was estimated to be 1.0 ng.ml, except for D in CSF which was estimated to be 0.2 ng/ml.

#### Markers of Cellular Senescence and SASP:

The Mesoscale Discovery U-Plex Biomarker Group 1 (hu) 71-plex panel (MesoScale Discovery, Natickm MA) was used to measure IL-6, a prespecified secondary outcome, and additional cytokines and chemokines in CSF and plasma. Samples were diluted and measured in duplicate, as per the manufacturer’s protocol. A MESO QuickPlex SQ 120MM instrument was used to measure the concentration of each marker.

#### ADRD Biomarkers:

A Simoa HD-X analyzer (Quanterix, Lexington, MA) was used to measure phosphorylated tau (p-Tau) 181 (SIMOA pTau-181 Advantage V2 kit, Quanterix, Lexington, MA), Aβ40, Aβ42, glial fibrillary acidic protein (GFAP), and neurofilament light (NFL) (SIMOA Neuro 4-Plex E Advantage kit, Quanterix, Lexington, MA) concentrations in plasma and CSF. The SIMOA pTau-231 Advantage kit (Quanterix, Lexington, MA) was used to measure pTau 231 concentration in CSF. Prior to loading the samples onto the Simoa analyzer, plasma and CSF samples were clarified by centrifugation at 14,000 x *g* for 10 minutes. All samples were run in duplicate. In addition, a Fujirebio G1200 (Malvern, PA) was used to measure total tau (lumipulse G total tau, Malvern, PA), pTau-181 (lumipulse G pTau-181, Malvern, PA) Aβ40 (lumipulse G B-Amyloid 1–40, Malvern, PA), and Aβ42 (lumipulse G B-Amyloid 1–42, Malvern, PA) as per the manufacturer’s protocol.

### Statistical Analysis:

Descriptive analyses were performed on baseline demographic and broader sample characteristics. Baseline to post-treatment changes in safety labs, vitals and body mass index (BMI), cognitive and functional assessment, neuroimaging outcomes, and biofluid markers were assessed using paired samples t-tests. All analyses were performed using SPSS version 28.0. Statistical tests were 2-sided and statistical significance was set at p<0.05. Given the exploratory nature of the pilot study, p-values were not corrected for multiple comparisons unless otherwise noted in the text.

## Results:

### Participants:

**Figure 2:**
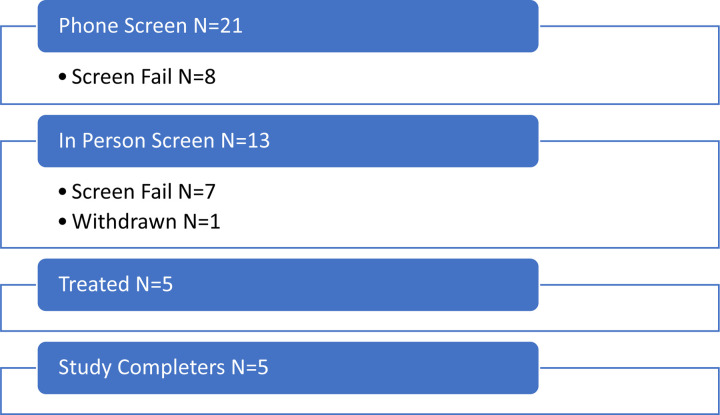
CONSORT Flow Diagram. Participant allocation in the open-label pilot study.

A total of 21 participants were screened over the phone, eight of whom did not meet the eligibility criteria (Figure 2). Thirteen participants completed the in-person screening visit and of those, seven were screen failures and one withdrew. Five participants (aged 70–82 years; median 76; 40% female; 80% Non-Hispanic White; 20% Hispanic) enrolled in the intervention. Regarding highest level of educational attainment, two participants (40%) had high school diplomas, one (20%) had some college, and two participants (40%) had college degrees or higher.

### Safety and Adherence:

A total of six adverse events (AEs) occurred during the course of the study, of which three (two mild: diarrhea and emesis, urinary tract infection, one moderate: hypoglycemia) occurred following the start of the intervention. The two mild AEs were deemed unlikely related to the study and the one moderate AE, hypoglycemia, was deemed possibly related to the intervention. Prior to the start of the intervention, there was one moderate severity AE (fall resulting in hematoma) and two mild AEs (hematuria, diarrhea). All AEs fully resolved within one to 16 days.

There were no significant changes in BMI (pre-treatment: 23.0±4.3 mg/k^2^; post-treatment: 22.7±4.2 mg/kg^2^, t(4)=−1.12, p=0.32), systolic blood pressure (pre-treatment: 114.4±11.8 mmHg; post-treatment: 120.4±16.7 mmHg, t(4)=0.56, p=0.61) or diastolic blood pressure (pre-treatment: 63.8±14.3 mmHg; post-treatment: 67.4±9.2 mmHg, t(4)=0.96, p=0.39).

There was a statistically significant, but not clinically significant, increase in total cholesterol from pre- to post-treatment (pre-treatment: 169.2±35.5 mg/dl; post-treatment: 179.4±40.0 mg/dl, t(4)=2.904, p=0.044). No other significant changes in safety lab parameters were observed (Supplementary Table 1).

All five participants who enrolled in the intervention completed the trial with a 100% study drug adherence rate.

### Study Drug Concentrations:

**Figure 3:**
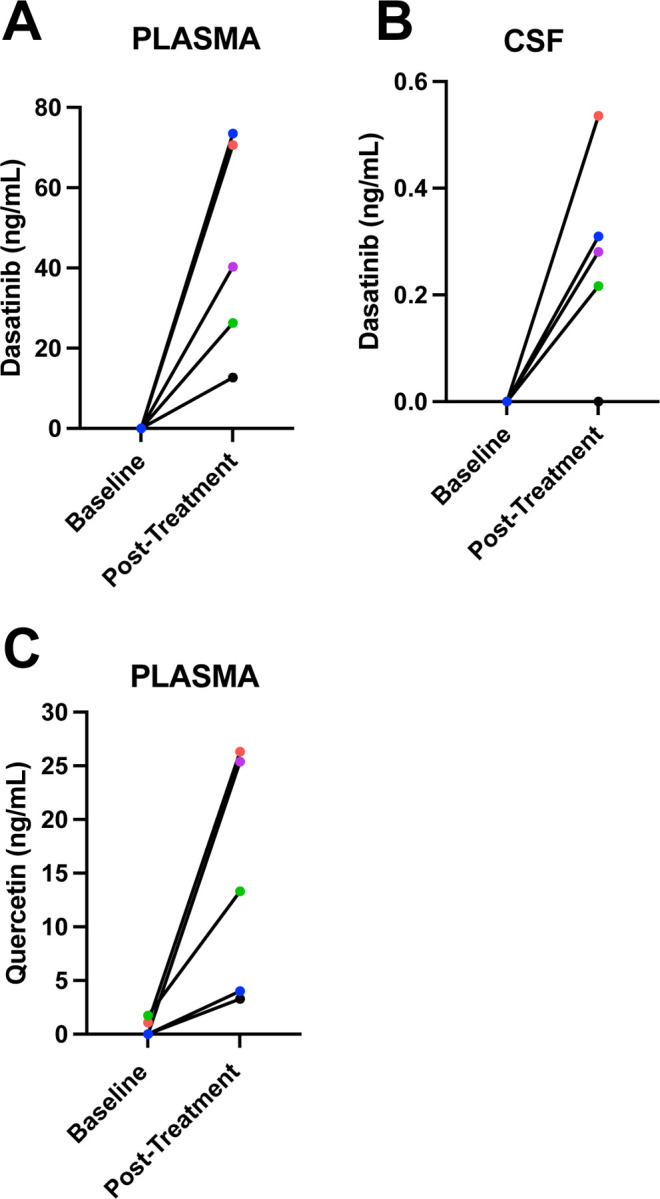
Concentration of D (Post-Treatment) and Q (Pre- and Post-Treatment) concentrations in blood and CSF quantified by High Performance Liquid Chromatography (HPLC) with Tandem Mass Spectrometry detection (MS/MS) method (HPLC/MS/MS).

As expected, D was not present in plasma or CSF prior to treatment (Figure 3A-B). After the intervention, D was detected in plasma in all five participants, ranging from 12.7 to 73.5 ng/ml. In CSF, post-treatment D levels were slightly above the LOQ (0.2 ng/ml) in four out of five participants, ranging from 0.281 to 0.536 ng/ml, and the fifth had no detectable levels. In the four participants with detection of D in CSF, the CSF to plasma ratio of D concentrations ranged from 0.004 to 0.008. D metabolites were undetected in both plasma and CSF with the exception of one post-treatment plasma specimen, 1.94 ng/ml (LOQ 1.0 ng/ml; metabolite data not shown).

Q is found in many fruits and vegetables^42,43^. In plasma at baseline, three participants had no detectable Q levels and the other two had concentrations of 1.09 and 1.73 ng/ml (LOQ 1.0 ng/ml). However, the two participants that had Q levels just above the LOQ at baseline had Q concentrations of 26.3 and 13.3 ng/ml post-treatment. Following treatment, Q was detected in plasma across participants, ranging from 3.29–26.30 ng/ml (Figure 3C). Within CSF, Q was not detected either before or after treatment across participants. Q metabolites were detected only in two post-treatment plasma specimens, 2.92 and 3.80 ng/ml (LOQ 1.0 ng/ml) and one post-treatment CSF sample, 1.23 ng/ml (LOQ 1.0 ng/ml; metabolite data not shown). Within CSF, Q was not detected either before or after treatment across participants.

### Cognitive and functional outcomes:

Baseline to post-treatment changes in the pre-specified cognitive outcomes, MoCA and CDR SOB, were not significant (Table 1). There was a statistically significant decrease on HVLT-R Immediate Recall. All other cognitive tests, as well as questionnaires assessing neuropsychiatric symptoms and functional status, did not demonstrate any significant changes.

### Neuroimaging:

Paired t-tests of pre- versus post-treatment MRIs revealed no significant differences in total brain volume, gray matter or white matter density, or right or left hippocampal volume, indicative of stable brain morphology over the three-month assessment period (Table 2).

### Markers of Cellular Senescence and SASP:

Applying the unadjusted p<0.05 cut-off, plasma levels of IL-17E, IL-21, IL-23, IL-17A/F, IL-17D, IL-10, VEGF, IL-31, MCP-2, MIP-1β and MIP-1α decreased from pre- to post-treatment, whereas YKL-40 levels increased. In CSF, TARC, IL-17A, I-TAC, Eotaxin-2, Eotaxin, and MIP-1α levels decreased, and IL-6 levels increased from pre- to post-treatment (Table 3

### ADRD Biomarkers:

**Figure 4:**
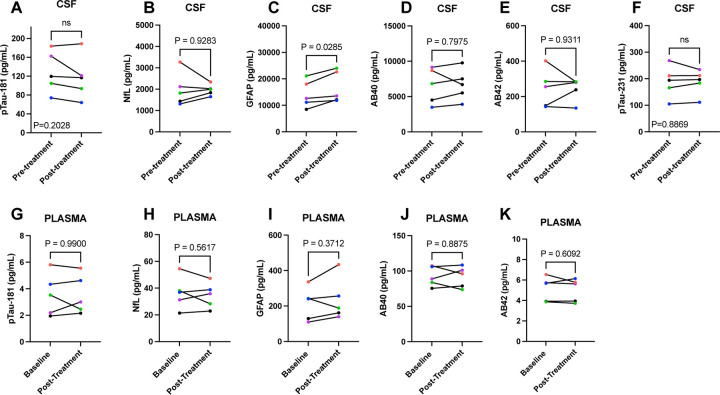
Baseline and Post-Treatment Alzheimer’s Disease and Related Dementia Plasma and Cerebrospinal Fluid Biomarkers Assessed Using the Simoa HD-X Analyzer. Values derived from paired samples t-test and p-value of 0.05.

**Figure 5:**
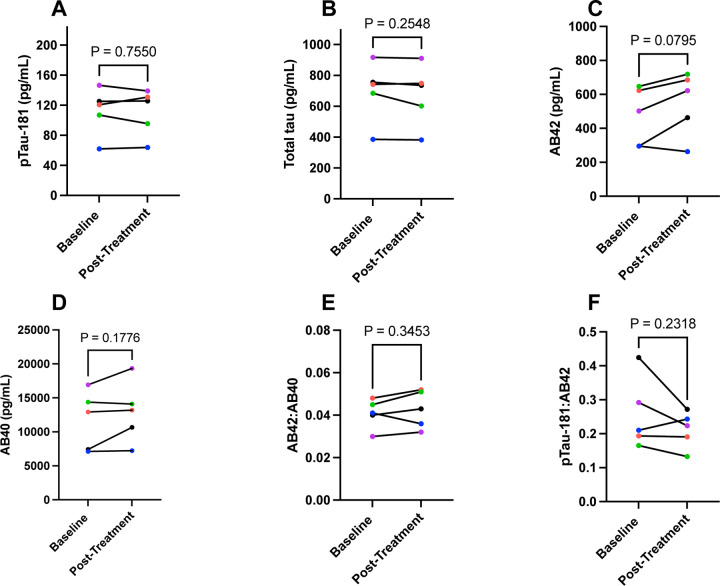
Baseline and Post-Treatment Alzheimer’s Disease and Related Dementia Cerebrospinal Fluid Biomarkers Assessed Using the Lumipulse. Values derived from paired samples t-test and p-value of 0.05

Using the SIMOA assays, there were no pre- to post-treatment changes in plasma or CSF protein levels with the exception of a significant increase of GFAP levels in CSF (Figure 4A-K). For the Lumipulse assays, no significant treatment changes were observed in CSF; however, there was a trend (p=0.0795) towards higher Aβ42 levels post-treatment (Figure 5A-F).

## Discussion

Cellular senescence has been associated with neurodegenerative disease in human pathology studies and preclinical models^6,7,18,19^. Herein, we present the results of the first-in-human trial of senolytic therapy for AD^28^. The primary aim of our open-label pilot study was to evaluate the CNS penetrance of first-generation senolytics, D and Q. Our results confirmed the presence of D in CSF following treatment. In addition, the intervention was well-tolerated with no premature discontinuation and only three AEs occurring following treatment initiation. Our study was not designed or powered to detect efficacy. However, our preliminary data suggests the potential of baseline to post-treatment changes in markers of cellular senescence and ADRD, which will require further exploration and validation in randomized placebo-controlled trials that are presently underway (NCT04685590).

A primary challenge to conducting trials for AD and other neurological diseases is the determination of the appropriate drug dosing as assessing pharmacokinetics in the CNS is highly invasive. In our study, we selected the combination of D and Q as they are among the best characterized senolytic agents, target multiple SCAP pathways, and are repurposed^8,25^, expediting clinical testing. The doses and intermittent scheduling regimen were selected based on prior research demonstrating safety and early indications of efficacy for other disease indications^8,26^. The intermittent dosing regimen was implemented because senescent cells across organ systems, including the brain, typically accumulate over a period of weeks, suggesting that drugs do not continually need to be present to be effective^6,21^. Intermittent dosing further reduces potential toxicity. Our study design of in-clinic administrations on the first day of each drug cycle enabled us to carefully monitor participant safety and was likely supportive of our 100% study drug adherence rate. In plasma, D has been shown to reach peak concentrations within two hours of administration^44^; however, the absorption in the CNS is less well established. Following oral ingestion of D in mice, a prior study reported D in brain homogenates using HPLC/MS at concentrations that were 12- to 31-fold lower than in plasma^44^. In humans, D has demonstrated efficacy for treating ALL and CML with CNS involvement and responses can be maintained for months to years^44^, suggesting a robust CNS treatment effect. However, HPLC/MS studies conducted in CSF taken from D-treated individuals with CML or ALL have reported low CSF concentrations and high variability across individuals^44,45^. Gong et al. examined plasma and CSF concentrations of D among individuals with ALL approximately two hours after a single dose of 100 mg of D^45^. Detectable D levels in CSF were only observed in 16% of participants (4/25 individuals) with ranges between 0.23 to 0.68 ng/ml. In our study, we observed a similar range of D concentrations in CSF. However, the detectable levels were more readily observed in our population, occurring in 80% (4/5 individuals) of participants. More consistent CSF concentrations may have been observed in our study of individuals with AD due to the disease’s impact on blood brain barrier integrity^46^. Future pharmacokinetic studies will be helpful for informing on the optimal dosing for desired CNS effects. However, our study demonstrated that D penetrated the CNS and prior research in oncology has shown that the medication can demonstrate CNS efficacy at low or even subnanomolar concentrations^44,47^.

In our study, Q was consistently detected in plasma across participants. However, unlike D, Q was not detectable in CSF within our sample. In animal model research, oral administration of Q has been shown to reduce oxidative stress in the brain^48,49^, suggesting a therapeutic effect in the CNS. In a preclinical study of mice that ingested 21.3 grams of Q per day, Q was detectable in brain homogenates assessed using HPLC-tandem mass spectrometry, plateauing after one-week of administration^49^. In culture, Q has been shown to permeate primary brain microvessel endothelial cells and primary astroglia cells^50^, suggesting blood brain barrier penetrance. However, confirmatory studies in humans are lacking. Q is rapidly metabolized in the human intestinal mucosa and liver and it has low bioavailability^51^, which may explain why it was not detectable in CSF within our study. There are ongoing efforts to improve the CNS permeability with the use of nanoparticles and/or chemical modification^49^. Further pharmacokinetic studies of Q in humans are warranted.

As the first-in-human clinical trial of senolytic therapy for AD, our study also provides important preliminary data on safety, tolerability, and feasibility. Throughout the study, a total of six AEs occurred, of which three emerged after treatment initiation. Two of these AEs were mild and highly common in the study population. Hypoglycemia was observed in one participant. D has been associated with changes in glucose regulation with reports of both hyper- and hypoglycemia emerging^52,53^. It has been hypothesized that the responses may differ depending on age, genetics, and comorbidity burden^53^. Without larger sample sizes and a placebo group, we are unable to determine if hypoglycemia occurred more frequently in the active treatment arm. Regular assessments of glucose levels in future trials may be helpful for further clarification. Clinical safety labs were generally stable from baseline to post-treatment. Only one statistically significant change emerged, which was an increase in total cholesterol levels. However, cholesterol levels remained in the normative range. A prior retrospective study conducted in adults with CML and normal baseline glucose-lipid levels suggested that D may cause mild increases in glucose, triglyceride and LDL-cholesterol levels^53^. In contrast to the typical treatment of CML, the intermittent dosing approach used in this trial may have helped to attenuate metabolic changes.

Our study was not powered to examine target engagement, but instead designed to collect exploratory data on baseline to post-treatment changes in markers of cellular senescence and SASP both in CSF and blood. Change in IL-6 was a prespecified secondary outcome. The analyses revealed a statistically significant elevation of IL-6 in CSF after treatment. Plasma levels modestly increased, but did not reach statistical significance. The treatment-induced changes in IL-6 may reflect senescent cell apoptosis whereby IL-6 was directly released from senescent cells upon their lysis; alternatively, apoptosis may have initiated an immune response to clear the cellular debris. Recognizing that IL-6 is a pleiotropic cytokine, we simultaneously performed a broader evaluation of cytokines and chemokines to better infer the treatment effect. CSF analyses indicated baseline to post-treatment decreases in adaptive immunity markers, TARC, IL-17A, I-TAC, Eotaxin and Eotaxin-2; and chemokine, MIP-1α. A similar pattern was observed in plasma whereby treatment was associated with a decrease in adaptive immunity markers IL-23, IL-21, IL-17, IL-31, and VEGF^54^; and chemokines, MIP-1α and MIP-1β. Given that senescent cells secrete these molecules as SASP factors, the observed reduction support a decrease in senescent cell burden post-treatment. While the majority of markers displayed reductions from pre- to post-treatment, there was variability. It is important to highlight that none of the markers would have withstood multiple comparisons correction, and the preliminary findings require further replication in studies designed to assess this endpoint.

Consistent with AD trials, our study also acquired cognitive and neuroimaging measures. Baseline to post-treatment changes were not observed for our pre-specified cognitive endpoints, the MoCA and CDR SOB. The null findings are not surprising as our trial was not designed to evaluate efficacy and included a small sample size and short duration of treatment. Prior studies in AD suggest that study durations of 18-months are required to observe decline in placebo groups^55^, providing a framework for trial lengths to assess efficacy. In our exploratory assessment of the broader cognitive battery, baseline to post-treatment performances were stable. There was a statistically significant decrease on a verbal learning measure (HVLT-R), however, without a control group, we are unable to compare the findings relative to the natural neurodegenerative disease course. Regarding neuroimaging outcomes, there were not significant changes in total brain volume, hippocampal volume, or gray matter or white matter density from baseline to post-treatment. While our study was underpowered and of insufficient duration to provide a comprehensive evaluation of neuroimaging outcomes, we consider the absence of changes to indicate a favorable safety profile of senolytic treatment. The data further underscore the need for randomized clinical trials designed to evaluate these metrics.

As a secondary outcome, our study also evaluated key ADRD biomarkers in both plasma and CSF at baseline and post-treatment. There were no significant changes in plasma biomarkers, which was anticipated given the small sample size and short follow-up period. In CSF, we observed a significant increase in GFAP levels from baseline to post-treatment. CSF GFAP levels are presumed to reflect reactive astrogliosis^56^ and demonstrate elevations early in the neurodegenerative disease process^57^. In our study, it is unclear if increases in GFAP reflect or an acute response to treatment. Coupled with the elevated CSF IL-6 data, it is tempting to speculate that the concomitant increase in GFAP may reflect apoptosis of senescent astrocytes. Supporting evidence for this would require additional blood and CSF collections, weeks or months after the end of treatment, to determine if increased GFAP and IL-6 were transient or sustained responses to senolytic treatment. Our preclinical trial of D+Q reported 35% fewer insoluble NFTs in the treatment arm relative to placebo^6^, which may have reflected a reduction in tangle formation and/or an increase in tau clearance. In our study, we did not observe changes in total tau, p-tau-181, or p-tau-231, however, the study was not powered to assess these outcomes. On-going efforts by our team are focused on a more comprehensive analyses of phospho-tau in CSF and post-mortem human brain to identify which tau species best reflect senescence. The results from the Lumipulse assay, but not from the SIMOA assay showed a trend towards increased post-treatment Aβ42 levels. If replicated in well-powered studies designed to assess efficacy, the findings could suggest the possibility of disease modification with senolytic treatment.

While our study provides the first report of senolytic treatment in humans with AD, there are several important limitations that must be considered. First, our study was designed to evaluate the CNS penetrance of D and Q. Therefore, it was not powered to assess outcomes related to target engagement, cognition, or disease modification. The short trial duration and lack of a placebo group place further restrictions on interpreting these outcomes. Another limitation is the lack of established senescence and SASP markers related to AD. Prior studies have reported that biomarkers of cellular senescence vary significantly across cell types and inducers^58,59^. Therefore, further work is necessary to identify clinically meaningful markers of cellular senescence in AD across specimen types, and is under investigation by our team. Our exploratory findings provide initial data on changes in protein levels following senolytic treatment in older adults with AD, but validation and replication in well-powered randomized controlled studies are critical for advancing therapeutic discovery in the field.

In summary, we report findings from the first clinical trial of senolytic therapy for AD. In alignment with our primary study aim, we identified support for the CNS penetrance of D, although Q was not detectable in CSF. In our study, the treatment was well-tolerated with excellent adherence to the study drug regimen. Broader assessments of target engagement and treatment-related outcomes were assessed to provide early feasibility data. While our study was not designed to evaluate efficacy, the data suggests the potential of treatment-related changes in markers of cellular senescence and AD pathology. Our vanguard study provides initial data on the safety, tolerability, and feasibility of senolytic therapy for AD. While early results are promising, fully powered, double-blinded, placebo-controlled studies are needed to evaluate the safety and potential for disease modification with the novel approach of targeting cellular senescence in AD.

## Supplementary Material

Supplement 1

## Figures and Tables

**Table 1:** Baseline and Post-Treatment Cognitive and Functional Status Assessments

Cognitive Test	Baseline Mean (SD)	Post-Treatment Mean (SD)	T-test(df), p-value
MoCA	16.2 (2.9)	16.0 (1.1)	t(4)=−0.196, p=0.85
CDR Sum of Boxes	5.30 (2.2)	5.60 (2.0)	t(4)=2.449, p=0.070
HVLT-R Immediate Total Recall	13.80 (4.4)	10.20 (4.6)	t(4)=−3.674, p=0.021*
HVLT-R Delayed Recall	0.60 (0.9)	0.40 (0.9)	t(4)=−1.000, p=0.37
WMS Logical Memory Immediate Recall	12.6 (6.5)	13.2 (1.9)	t(4)=0.220, p=0.84
WMS Logical Memory Delayed Recall	14.2 (2.2)	12.0 (2.8)	t(4)=−1.633, p=0.18
Benson Figure Copy	8.60 (6.2)	14.6 (2.5)	t(4)=2.390, p=0.075
Benson Figure Delayed Recall	0.80 (1.5)	2.50 (3.1)	t(4)=2.049, p=0.13
Number Span Forward	6.40 (1.8)	6.80 (1.6)	t(4)=0.784, p=0.48
Number Span Backward	4.60 (1.7)	4.80 (1.3)	t(4)=0.343, p=0.75
Trails A, Time to Completion (Seconds)	76.0 (35)	99.0 (71)	t(4)=1.137, p=0.32
Trails B, Time to Completion (Seconds)	208 (86)	212 (84)	t(4)=0.829, p=0.45
Phonemic Fluency (F,A,S)	32.0 (5.8)	31.4 (5.5)	t(4)=−0.187, p=0.86
Semantic Fluency (Animals)	9.80 (1.3)	11.0 (3.2)	t(4)=1.124, p=0.32
Lawton IADL	11.0 (4.9)	10.4 (5.3)	t(4)=−0.612, p=0.57
GDS-15	4.00 (3.1)	3.60 (2.4)	t(4)=−0.459, p=0.67
NPI	5.40 (7.7)	3.80 (4.8)	t(4)=−0.758, p=0.49

Note: Baseline to post-treatment changes were assessed using paired samples t-tests. MoCA = Montreal Cognitive Assessment, CDR = Clinical Dementia Rating scale, HVLT-R = Hopkins Verbal Learning Test Revised, WMS = Weschler Memory Scales, Trails = Trail Making Test, IADL = Independent Activities of Daily Living, ADL = Activities of Daily Living, GDS-15 = Geriatric Depression Scale 15-Item, NPI = Neuropsychiatric Inventory,

* p<0.05

**Table 2:** Baseline and Post-Treatment Neuroimaging Outcomes

Brain Region (voxels)	Baseline Mean (SD)	Post-Treatment Mean (SD)	T-test(df), p-value
Intracerebroventricular Volume (ICV)	1393962.33 (151287.55)	1393461.27 (149379.55)	t(3)=0.150, p=0.89
Total Brain Volume/ICV	0.856 (0.005)	0.851 (0.009)	t(3)=1.732, p=0.18
Grey Matter Volume/ICV	0.359 (0.021)	0.359 (0.015)	t(3)=0.522, p=0.64
White Matter Volume/ICV	0.452 (0.006)	0.446 (0.009)	t(3)=1.192, p=0.32
Right Hippocampus Volume/ICV	0.002 (0.00016)	0.0002 (0.00013)	t(3)=0.472, p=0.67
Left Hippocampus Volume/ICV	0.002 (0.00008)	0.002 (0.00012)	t(3)=0.313, p=0.77

Note: Baseline to post-treatment changes were assessed using paired samples t-tests. Brain regions normalized to intracerebroventricular volume (IVC) measured in voxels, p<0.05.

**Table 3:** Significantly Differentially Expressed Proteins in Plasma and Cerebrospinal Fluid from Baseline to Post-Treatment

Protein (pg/mL)	Baseline Mean (SD)	Post-Treatment Mean (SD)	Fold Change	T-test(df), p-value
**Plasma**				
*IL6	1.30 (0.54)	1.58 (0.81)	1.22	t(4)= 1.651, p=0.145
IL-17E	25.2 (20.3)	17.0 (14.5)	0.67	t(4)= −5.216, p=0.0014
IL-21	166 (96.7)	102 (72.1)	0.62	t(4)=−4.714, p=0.002
IL-23	53.4 (29.5)	34.0 (25.4)	0.64	t(4)=−4.345, p=0.003
IL-17A/F	35.4 (12.8)	23.1 (12.7)	0.65	t(4)=−3.870, p=0.007
IL-17D	67.7 (25.5)	49.0 (17.9)	0.72	t(4)=−3.384, p=0.013
IL-10	0.31 (1.0)	0.21 (0.20)	0.66	t(4)=−3.347, p=0.013
VEGF	20.8 (6.0)	12.5 (3.4)	0.60	t(4)=−3.238, p=0.015
YKL-40	58047 (60143)	105444 (127256)	1.82	t(4)=3.017, p=0.020
IL-31	67.5 (27.9)	54.8 (28.9)	0.81	t(4)=−2.914, p=0.024
MCP-2	23.5 (5.1)	18.5 (4.5)	0.79	t(4)=−2.806, p=0.028
MIP-1β	48.5 (16.1)	36.9 (25.8)	0.76	t(4)=−2.515, p=0.042
MIP-1α	24.3 (1.6)	19.6 (4.7)	0.81	t(4)=−2.433, p=0.047
**Cerebrospinal Fluid**				
*IL-6	1.16 (0.32)	1.55 (0.23)	1.34	t(4)=3.913, p=0.008
TARC	1.42 (0.42)	1.25 (0.38)	0.87	t(4)=−3.099, p=0.021
IL-17A	0.54 (0.13)	0.35 (0.11)	0.60	t(4)=−2.753, p=0.033
I-TAC	4.15 (1.2)	3.36 (1.1)	0.81	t(4)=−2.736, p=0.033
Eotaxin-2	14.5 (6.5)	12.8 (5.3)	0.89	t(4)=−2.630, p=0.038
Eotaxin	16.9 (4.9)	14.7 (4.3)	0.87	t(4)=−2.534, p=0.044
MIP-1α	19.0 (2.4)	14.3 (4.8)	0.75	t(4)=−2.471, p= 0.048

Note: Differential expression analysis was carried out by the moderated t-test

*: Prespecified secondary outcome

IL = interleukin, MIP = Macrophage Inflammatory Protein, G-CSF = Granulocyte Colony-Stimulating Factor, TRAIL = Tumor Necrosis Factor Related Apoptosis-Inducing Ligand, TARC = Thymus- and Activation-Regulated Chemokine, p<0.05
