# Supplementary material for: Senolytic therapy to modulate the progression of Alzheimer’s Disease (SToMP-AD) – Outcomes from the first clinical trial of senolytic therapy for Alzheimer’s disease
Source: Res Sq. 2023 Apr 24:rs.3.rs-2809973. Preprint. [Version 1] doi: 10.21203/rs.3.rs-2809973/v1 (PMC10168460; doi:10.21203/rs.3.rs-2809973/v1)
Supplement: Supplement 1 [file NIHPPRS2809973V1-supplement-1.pdf]

Supplementary Table 1: Baseline and Post-Treatment Safety Labs

**Blood Marker**	**Baseline Mean (SD)**	**Post-Treatment Mean (SD)**	**T-test(df), p-value**
White blood cells (K/uL)	7.0 (2.3)	5.8 (1.8)	t(4)=-1.197, p=0.30
Red blood cells (MIL/uL)	4.5 (0.7)	4.5 (0.8)	t(4)=-0.726, p=0.51
Hemoglobin (g/dl)	14.0 (1.7)	14.0 (1.8)	t(4)=-0.215, p=0.84
Hematocrit (%)	41.8 (5.)	41.1 (6.6)	t(4)=-0.497, p=0.65
MCV (fL)	92.2 (2.7)	92.6 (2.7)	t(4)=0.667, p=0.54
MCH (pg)	31.0 (1.3)	31.6 (1.8)	t(4)=1.199, p=0.30
MCHC (g/dl)	33.6 (1.8)	34.2 (1.4)	t(4)=0.756, p=0.49
RDW (%)	12.6 (0.6)	12.6 (0.5)	t(4)=-0.279, p=0.79
Platelets (K/uL)	224.0 (69.5)	235.8 (77.8)	t(4)=0.717, p=0.51
Absolute Neutrophils (K/uL)	69.0 (6.6)	64.4 (2.2)	t(4)=-1.696, p=0.17
Absolute Lymphocyte (K/uL)	19.0 (6.2)	22.2 (4.8)	t(4)=1.536, p=0.20
Absolute Monocyte (K/uL)	8.8 (1.9)	9.8 (2.8)	t(4)=1.826, p=0.14

Absolute Eosinophil (K/uL)	2.2 (0.8)	2.2 (0.5)	t(4)<0.001, p=1.0
Absolute Basophil (K/uL)	0.8 (0.5)	1.0 (0)	t(4)=1.000, p=0.37
Glucose (mg/dL)	91.4 (14.6)	87.6 (24.2)	t(4)=-0.656, p=0.55
Blood Urea Nitrogen (mg/dL)	17.6 (6.5)	16.0 (1.7)	t(4)=-0.726, p=0.51
Creatinine (mg/dL)	0.90 (0.15)	0.86 (0.15)	t(4)=-0.539, p=0.62
eGFR (mL/min/1.73)	73.2 (10.6)	79.6 (9.6)	t(4)=1.082, p=0.34
Sodium (mmol/L)	140.4 (1.5)	141.0 (2.1)	t(4)=0.440, p=0.68
Potassium (mmol/L)	4.3 (0.3)	4.2 (0.3)	t(4)=-0.250, p=0.81
Calcium (mg/dL)	9.5 (0.5)	9.4 (0.5)	t(4)=-1.725, p=0.16
Total Protein (g/dL)	6.7 (0.5)	6.6 (0.4)	t(4)=-2.359, p=0.078
Albumin (g/dL)	4.3 (0.2)	4.2 (0.2)	t(4)=-1.000, p=0.37
Bilirubin (mg/dL)	0.6 (0.3)	0.4 (0.1)	t(4)=-2.236, p=0.089
Alkaline Phosphate (IU/L)	73.2 (23.7)	75.2 (23.7)	t(4)=0.381, p=0.72
Aspartate Aminotransferase (IU/L)	21.2 (6.2)	21.6 (4.2)	t(4)=0.209, p=0.84
Alanine Transaminase (IU/L)	15.6 (3.6)	17.0 (5.2)	t(4)=0.560, p=0.61
Total Cholesterol (mg/dL)	169.2 (35.5)	179.4 (40.0)	t(4)=2.904, p=0.044*
Triglycerides, (mg/dL)	79.6 (9.4)	102.0 (13.5)	t(4)=2.535, p=0.064

HDL-Cholesterol (mg/dL)	68.6 (20.0)	67.6 (21.7)	$t(4)=-0.632$, $p=0.56$
LDL-Cholesterol (mg/dL)	85.5 (17.9)	93.8 (21.9)	$t(4)=2.493$, $p=0.067$
Hemoglobin A1c (%)	5.4 (0.4)	5.3 (0.2)	$t(4)=-0.739$, $p=0.50$

*$p<0.05$
